# Critical Redistribution of Nitrogen in the Austenitic Cr-Mn Steel under Severe Plastic Deformation

**DOI:** 10.3390/ma14237116

**Published:** 2021-11-23

**Authors:** Valery Shabashov, Kirill Lyashkov, Kirill Kozlov, Vladimir Zavalishin, Andrey Zamatovskii, Natalya Kataeva, Victor Sagaradze, Yurii Ustyugov

**Affiliations:** 1 M.N. Mikheev Institute of Metal Physics, Ural Branch, Russian Academy of Sciences, 620108 Ekaterinburg, Russia; lyashkov@imp.uran.ru (K.L.); kozlov@imp.uran.ru (K.K.); zavali@imp.uran.ru (V.Z.); zamatovsky@imp.uran.ru (A.Z.); kataeva@imp.uran.ru (N.K.); vsagaradze@imp.uran.ru (V.S.); ustyugov@imp.uran.ru (Y.U.); 2 Institute of Engineering Science, Ural Branch, Russian Academy of Sciences, 620108 Ekaterinburg, Russia

**Keywords:** nitrogen, severe plastic deformation, mechanical alloying, Mössbauer spectroscopy

## Abstract

A narrow temperature range of changes in the mechanism and kinetics of structural-phase transformations during mechanical alloying under deformation in rotating Bridgman anvils was determined by the methods of Mössbauer spectroscopy, electron microscopy, and mechanical tests in the high-nitrogen chromium-manganese steel FeMn_22_Cr_18_N_0.83_. The experimentally established temperature region is characterized by a change in the direction of nitrogen redistribution—from an increase in the N content in the metal matrix during cold deformation to a decrease with an increase in the temperature and degree of severe plastic deformation. The change in the direction of nitrogen redistribution is due to the acceleration of the decomposition of a nitrogen-supersaturated solid solution of austenite with the formation of secondary nanocrystalline nitrides. The presence of a transition region for the mechanism of structural-phase transitions is manifested in the abnormal behavior of the mechanical properties of steel.

## 1. Introduction

High-nitrogen chromium-manganese austenitic steels have a number of favorable physical and chemical properties (corrosion resistance, tribological characteristics, etc.), which allow for offering these steels as new materials in mechanical engineering [1,2,3,4,5]. The mechanical properties of nitride steels are largely determined by structural-phase transformations during various treatments [5,6,7]. This applies, first of all, to transformations in conditions of intense severe plastic deformation (SPD), such as impact and friction loading, extrusion, equal-channel angular pressing (ECAP), high pressure torsion (HPT), etc. In [8,9], attention was paid to the processes of the dissolution of CrN (and Cr_2_N) nitrides, which was induced by HPT and sliding friction in the aging high-nitrogen austenitic steels FeMn_22_Cr_18_N_0.83_ and FeCr_22_Mn_1.4_N_1.24_. The carbon content in these steels was 0.05 and 0.08 wt%, respectively. In the austenitic steel FeMn_22_Cr_18_N_0.83_, the authors attribute the improvement of wear resistance characteristics, in particular, to an increase in hardness due to the solid-solution mechanism of friction hardening [8]. The high chemical activity and diffusive mobility of nitrogen can contribute to (i) the relaxation processes of nitride dissolution and (ii) the formation of secondary nitrides under SPD conditions [10]. An indirect confirmation for the formation of secondary nitrides in the austenite matrix can be presented by the transmission electron microscopy (TEM) data—on the formation of nanocrystalline nitrides—concerning high-nitrogen chromium-manganese steels under conditions of HPT deformation at room temperature and during processing in ball mills [9,10].

It has been shown in the works on SPD that a change in the temperature of large plastic deformation in a relatively narrow temperature range radically changes the direction of structural-phase transformations—from the disordering and dissolution of particles at temperatures near room (conditionally at “cold” deformation) to the accelerated ordering and formation of secondary phases at temperatures above room (conditionally at “warm” deformation) [11,12]. The “drastic” influence of temperature at SPD on the mechanism and kinetics of the structural-phase transitions of the type of mechanical alloying was shown in experiments on the deformation in rotating Bridgman anvils affecting the Fe–Ni–*Me* (Al, Ti) substitution alloys forming intermetallides [11,12] and carbon–supersaturated Fe–Ni–C interstitial alloys [11,13]. When milling in ball mills, the role of temperature in accelerating the processes of the ordering and formation of secondary phases is manifested due to local heating in zones of impact influence. Theoretically, such dynamic effects at *T*_ext.action_ ≤ 0.3*T*_melting_ are justified by the appearance of additional diffusion channels of relaxation of high mechanical energy at SPD [14]. Depending on the values of deformation parameters, various scenarios of non-equilibrium phase transitions are realized [15,16].

The aim of this work was the study of the influence of the SPD parameters (temperature and degree of deformation) on the mechanism and kinetics of structural-phase transitions, as well as on the mechanical properties of one of practically important austenitic alloys, namely, the aging high-nitrogen Cr–Mn austenitic steel.

## 2. Experimental

We investigated the high-nitrogen chromium-manganese austenitic steel FeMn_22_Cr_18_N_0.83_ (wt%: 0.05 C, 22.1 Mn, 17.9 Cr, 0.83 N, and Fe for the balance) prepared by the technology of casting with nitrogen back pressure [2]. Samples of the steel were homogenized at 1423 K, then subjected to hot forging, and finally quenched from 1423 K in water. Part of the quenched samples were subjected to artificial aging at 1073 K, for 2 h and 30 h. To carry out the SPD by the HPT method in a rotating Bridgman anvils, plates with a thickness of 0.3 mm were spark-cut off from rods with a cross section of 10 × 10 mm^2^ and thinned by mechanical grinding to 0.25 mm. Specimens of the steel in the quenched and the aged condition were deformed at temperatures of 298 K, 373 K, 473 K, 573 K, and 623 K, following the method of [11]. At a chosen temperature, specimens of steel were “loaded” by compression of 8.0 GPa, calculated as the force related to the anvil area. Then the deformation of the samples was carried out via rotating of the bottom anvil. After “shear”, a sample was “unloaded” and returned onto a room temperature. After HPT, the sample has acquired the form of a disk from 0.08 mm to 15 mm in thickness and 5 mm in diameter. The degree of deformation at HPT in the rotating Bridgman anvils can be estimated by the equation [17,18]:

(1) $$\varepsilon =\ln\left(h_{0}/h_{i}\right)+\ln{\left(1+{\left(\varphi R_{i}/h_{i}\right)}^{2}\right)}^{1/2},$$

where *h*_0_ and *h_i_* are the thickness of the samples before and after deformation, *φ* = *n·*2π is the angle of rotation of the anvils (*n* is the number of revolutions), *R_i_* is the distance from the center of the sample to the studied area of the structure (usually 1/2 of the sample radius). The deformation was carried out at a rotational speed of *ω* = 0.3 rpm. Note that Equation (1) is valid for small angles of rotation of anvils and does not take into account possible structural-phase transitions at SPD. For more complicated deformation effects, taking into account the nonlinearities of the deformation process and including elastic and plastic modes of deformation, the so-called Odquist parameter is used [19]. In the presented work, we investigated multistage structural-phase transformations in a metallic material. As a measure of the deformation effect, we used the number of revolutions of anvils *n* ranged from 0.5 to 5. The choice of the parameter *n* makes it possible to solve the posed experimental problem of structure evolution in a specific HPT deformation scheme. The structure evolution mentioned concerns (i) the redistribution of alloying elements, (ii) structural phase analysis, and (iii) mechanical properties of samples, depending on the number of revolutions of anvils *n* and temperature of HPT.

After deformation at different temperatures and the number of revolutions of anvils *n*, the micro hardness indentation (*H*_IT_) of the samples was studied on a Nanotest device (Micro Materials Ltd., Wrexham, UK) using a Berkovich indenter and using a pyramidal analysis [20]. Samples with a diameter of 5 mm were glued to the holder of the Nanotest device with cyanoacrylate-based glue and kept under pressure through a rubber gasket for at least an hour. The indentation procedure consisted in indentation of a Berkovich diamond indenter with a rate of increasing loading of 6.4 mN per second to a maximum load of 128 mN. Then, a holding for 10 s was following at a maximum load, and unloading—with the same rate. Based on the diagram of indentation, i.e., the dependence of the depth of indentation on the load, we calculated the values of hardness according to Berkovich using the Oliver–Pharr method of calculation [20,21]. Since the degree of deformation at HPT depends on the “radius of deforming”, for averaging, an area at the surface of a sample was chosen of a diameter of ~3 mm, on which 81 indentations were performed (on a square grid of 9 × 9 cells of linear dimensions 250 µm × 250 µm), which was followed by averaging of the measurement results. A moderate part of the results of indentations with obvious indications of a considerable deviation from the average value were excluded from consideration. The error of the average value *H*_IT_ was calculated as the mean-square-root deviation.

For the Mössbauer measurements, the samples were thinned to a thickness of 20 µm after mechanical tests. The Mössbauer absorption spectra of γ quanta with an energy of 14.4 keV at ^57^Fe nuclei were obtained at room temperature in the constant acceleration mode with a ^57^Co(Rh) source. The standard calibrator was the α-Fe foil. For the Mössbauer measurements, there was used either the full surface area of the sample or its ring area excluding the central part of radius 1 mm. The spectra in both of the cases were almost similar. The calculation of the Mössbauer spectra was carried out using the MS-Tools software package [22]. The calculation included the reconstruction of the distribution of the centers of gravity of the absorption lines on the scale of Doppler velocities *p*(*V*), which improve the resolution of the spectrum. Furthermore, the distribution of *p*(*V*), along with a priori information, was used to simulate and approximate the spectra by the sum of several subspectra corresponding to various non-equivalent surroundings of the Mössbauer probe, ^57^Fe atoms.

On the same samples, the structure was studied by TEM on a JEM-200CX microscope.

## 3. Results

Based on the posed aim of this work, the experiments on the deformation of the hardened and aged steel FeMn_22_Cr_18_N_0.83_ were performed at various degrees and temperatures of HPT.

### 3.1. TEM Analysis of the Results of HPT Deformation of the Steel FeMn_22_Cr_18_N_0.83_

**Figure 1:**
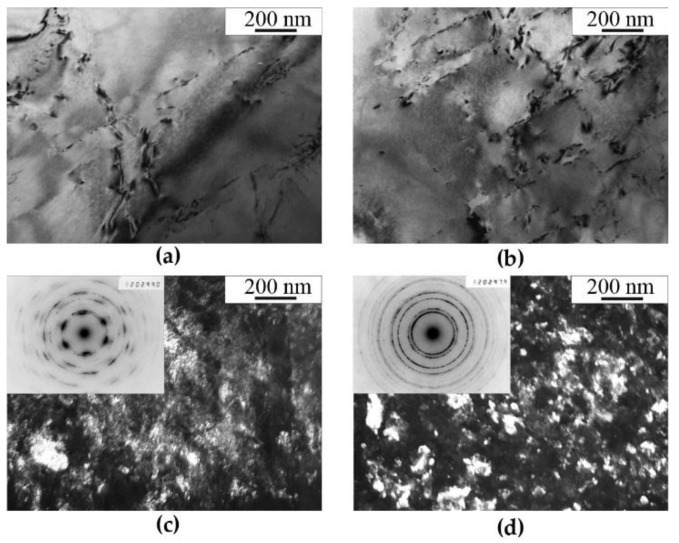
Structure of (**a**,**b**) quenched and (**c**,**d**) deformed FeMn_22_Cr_18_N_0.83_ steel. Treatment: (**a**,**b**) quenching; (**c**) quenching + HPT of *n* = 3 rev. at 298 K; (**d**) quenching + HPT of *n* = 3 rev. at 573 K. (**a**,**b**)—bright-field image, (**c**,**d**)—dark-field images in the combined reflection (111)γ + (200)γ + Cr_2_N.

Figure 1a,b shows a grained austenitic structure of the initial hardened steel, which does not contain visible nitride precipitates. As a result of HPT, a cellular structure is formed in the hardened steel (Figure 1c,d). Microdiffraction patterns show the azimuthal smearing of reflections, and not the very individual reflections themselves. An increase in the temperature of the HPT to 573 K leads to the formation of a submicrocrystalline structure (Figure 1c). A ring microdiffraction is also presented here, which contains reflections from highly misoriented austenite grains. The size of the matrix fragments (grains and subgrains) is 30 nm–100 nm.

**Figure 2:**
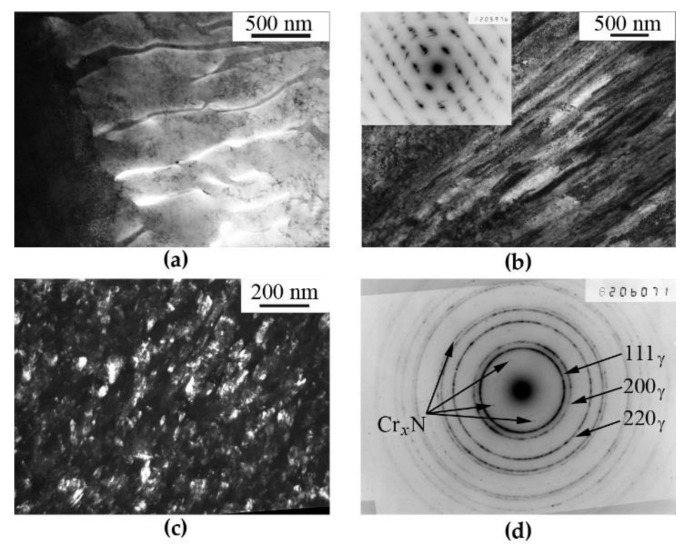
Structure of (**a**) aged and (**b**,**c**) deformed FeMn_22_Cr_18_N_0.83_ steel. Treatment: (**a**) quenching + aging at 1073 K, 2 h; (**b**) aging + HPT of *n* = 3 rev. at 298 K; (**c**) aging + HPT of *n* = 3 rev. at 573 K. (**a**,**b**)—Bright-field images, (**c**)—dark-field images in the combined reflection (111)γ + (200)γ + Cr_2_N. Electron-diffraction pattern (**d**) corresponding to the image (**c**).

Figure 2a shows the structure of FeMn_22_Cr_18_N_0.83_ steel after aging at 1073 K for 2 h. The cellular decomposition of austenite has occurred to ~20% of the sample volume. Shear under pressure at a temperature of 297 K entails the formation of a band structure (Figure 2b) in the regions where no cellular decomposition has occurred. In the microdiffraction pattern one can see the azimuthal smearing of reflections. In the regions where the cellular decomposition has occurred, one can observe in result of HPT the indications of occurrence of the fragmentation, partial dissolution, thinning of the plates of chromium nitrides.

As a result of HPT at 573 K, a submicrocrystalline structure is formed, Figure 2c. The size of fragments of the matrix (grains and subgrains) amounts to 30–50 nm. An electron diffraction pattern is also given here, which contains ring reflections of strongly misoriented austenite grains and separate reflections from chromium nitrides, which are located inside the first (111)_γ_ matrix ring, Figure 2d. In Figure 2c in an “austenite + chromium nitride” reflection (the result of reflection from planes with *d* ≈ 2.078Å) a dark-field image of secondary chromium nitrides up to 2 nm in size together with austenite fragments is given.

**Figure 3:**
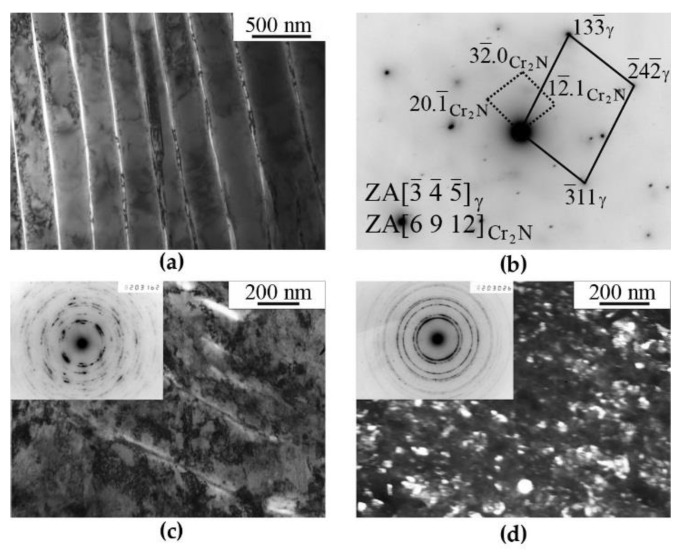
Structure of (**a**) aged and (**c**,**d**) deformed FeMn_22_Cr_18_N_0.83_ steel. Treatment: (**a**) quenching + aging at 1073 K, 30 h; (**c**) aging + HPT of *n* = 3 rev. at 298 K; (**d**) aging + HPT of *n* = 3 rev. at 573 K. (**a**,**c**)—bright-field images; (**d**)—dark-field images in the combined reflection (111)γ + (200)γ + Cr_2_N. Electron-diffraction pattern (**b**) corresponding to the image (**a**).

The aging of FeMn_22_Cr_18_N_0.83_ steel at 1073 K, for 30 h (15 times longer that the ageing before (of 2 h)) results in almost complete (~90%) decomposition of the solid solution with the formation of a perlite-like cellular structure, which presents by itself alternating plates of austenite and of chromium nitrides (Cr_2_N) [8], Figure 3a. HPT deformation performed at room temperature has led to the refining of the structure of austenite matrix and to a partial dissolution of nitrides, Figure 3c.

In the case of “warm” (573 K) deformation, the structure of quenched and aged steel is completely transformed in comparison to that of steel in the initial condition. No plate-like nitrides are preserved in the structure. Dispersed crystallites of the nitrides and austenite are formed. In the vicinity of the first diffraction ring of reflections from austenite matrix, the reflections that can be related to the nitrides of non-stoichiometric composition Cr*_x_*N are disposed, see Figure 1d, Figure 2c and Figure 3d. In the dark-field image, in the combined reflection (111)_γ_ + (200)_γ_ + Cr_2_N not only misoriented matrix fragments are shined, but also disperse precipitates with a size of ~2 nm. Nanocrystalline nitrides are likely to be secondary ones that have formed as a result of decomposition of the nitrogen-supersaturated solid solution based on the FCC phase. Note that TEM analysis was carried out mainly at a distance of ½ of the sample radius. TEM analysis showed that at *ε* > 4 (*n* ≥ 1), the type of submicrystalline structure does not depend much on *Ri*. The decomposition of the γ solid solution supersaturated with nitrogen and, in particular, the processes of a redistribution of nitrogen in the course of deformation-generating effect are confirmed by the data of Mössbauer spectroscopy. The results of Mössbauer-spectroscopy analysis are presented below.

### 3.2. Mössbauer-Spectroscopy Analysis of Nitrogen Redistribution in Austenite of the Steel FeMn_22_Cr_18_N_0.83_

**Figure 4:**
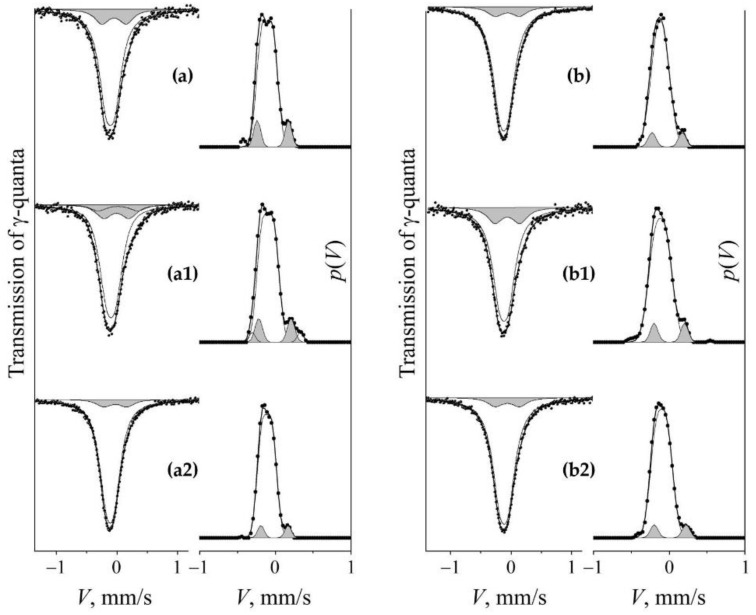
Mössbauer spectra and *p*(*V*) distributions for FeMn_22_Cr_18_N_0.83_ steel. Treatment: (**a**)—quenching; (**b**) aging at 1073 K, 30 h; (**a1**,**b1**)—HPT of *n* = 3 rev. at 298 K of quenched and 2-h aged steel, respectively; (**a2**,**b2**)—HPT of *n* = 3 rev. at 573 K of quenched and 2-h aged steel, respectively. The component of the “nitrogenous” doublet *S_D_*_1_ is highlighted by darkening.

The processes of mechanical alloying during the dissolution and precipitation of nitrides in the matrix of steel were expressed through a change in the nitrogen content in the solid solution of austenite. To analyze the nitrogen content using Mössbauer measurements, a model of the Mössbauer spectrum was used in the form of a superposition of the subspectra that are correspondent to the non-equivalent positions of nitrogen atoms in the nearest octahedral interstices (OIs) of the ^57^Fe resonance probe. The Mössbauer spectra of hardened and aged steel FeMn_22_Cr_18_N_0.83_ have the form of a broadened asymmetric singlet [8], see Figure 4. The calculation of the distribution of the intensity of the Mössbauer absorption on the Doppler velocity scale *p*(*V*) reveals the structure of the spectra. The approximation of *p*(*V*) by Gaussian distributions allows us for presenting the integral spectrum as a superposition of doublets *D*0 + *D*1 + *D*2. The *D*0 doublet is stipulated by the gradient of the electric field at the ^57^Fe core induced by the surroundings filled with substitution impurities (Mn, Cr), and its hyperfine parameters (isomer shift *I*_s_ and quadrupole shift *Q*_s_/2) are close to the values of the corresponding parameters of stainless steels [23,24]. *D*1 and *D*2 are doublets that have emerged due to the introduction of correspondingly one and two nitrogen atoms in the nearest OIs of resonant iron in the FCC crystal matrix. The hyperfine parameters of *D*1 and *D*2 are similar to the parameters of doublets in the spectrum of nitrided iron [25,26]. The proposed model of the spectra of the studied steel is confirmed by the results of experiments on the aging of steel, as well as the subsequent cold deformation of the HPT (see Figure 4). A similar result was obtained on this steel during processing by dry sliding friction [8]. A decrease in the relative intensity of doublets *D*1 and *D*2 during aging indicates the release of nitrogen from the interstitial solid solution into nitrides, and the increase in the intensity of doublets is associated with the dissolution of nitrogen in the austenite matrix during cold mechanical alloying initiated through HPT. X-ray diffraction analysis confirms a decrease in the nitrogen content in the FCC solid solution during artificial aging by recording the reduction in the lattice parameter from 0.3620 nm to 0.3614 nm. The doping of austenite with nitrogen after HPT (*n* = 3 rev.) at room temperature is proved by increasing the lattice period to 0.3648 nm.

The assessment of *c*_N_ nitrogen content in FeMn_22_Cr_18_N_0.83_ steel based on the use of the Mössbauer spectra was carried out by judging on the contribution *S_D_*_1_ of the configuration *D*1 from iron atoms with one nitrogen atom in the nearest octahedral interstices, where the *S_D_*_1_ being a relative integral intensity,—in accordance with the equation [25]:

(2) $$S_{D1}=6\cdot p\left(1-p\right),$$

where *p* = *c*_N_⋅(1 − *c*_N_) is the fraction of octahedral interstices (OIs) in austenite, with OIs occupied by nitrogen atoms. We performed calculation of the *S_D_*_1_ for the samples of the same thickness in the approximation of thin absorbing matter.

#### 3.2.1. Change of the Mössbauer Spectrum of Austenite Depending on the Deformation Temperature of the HPT of Quenched and Aged Steel

Figure 4 shows the spectra and distributions of *p*(*V*) of the initial samples of quenched (Figure 4a) and aged (Figure 4b) steel, as well as the results of their cold (298 K) deformation by HPT with *n* = 3 rev., see Figure 4a1 and Figure 4b1, respectively. “Loading” by HPT at 298 K of the aged steel causes a significant increase in the intensity of the “nitrogenous” doublet *S_D_*_1_ (from 10 to 15%), see Figure 4b1 and Table 1. When the quenched sample is loaded in the same way, the intensity of the *S_D_*_1_ doublet increases slightly in the spectrum and distribution of *p*(*V*), and an additional *S_D_*_2_ doublet emerges with an intensity of ~2–3%. The intensity growth of *S_D_*_1_ and the appearance of *S_D_*_2_ in the steel spectrum can be explained by an increase in the probability of mutually encountering between one and two nitrogen atoms in the sites of nearest surrounding of resonant iron [8,9].

**Figure 5:**
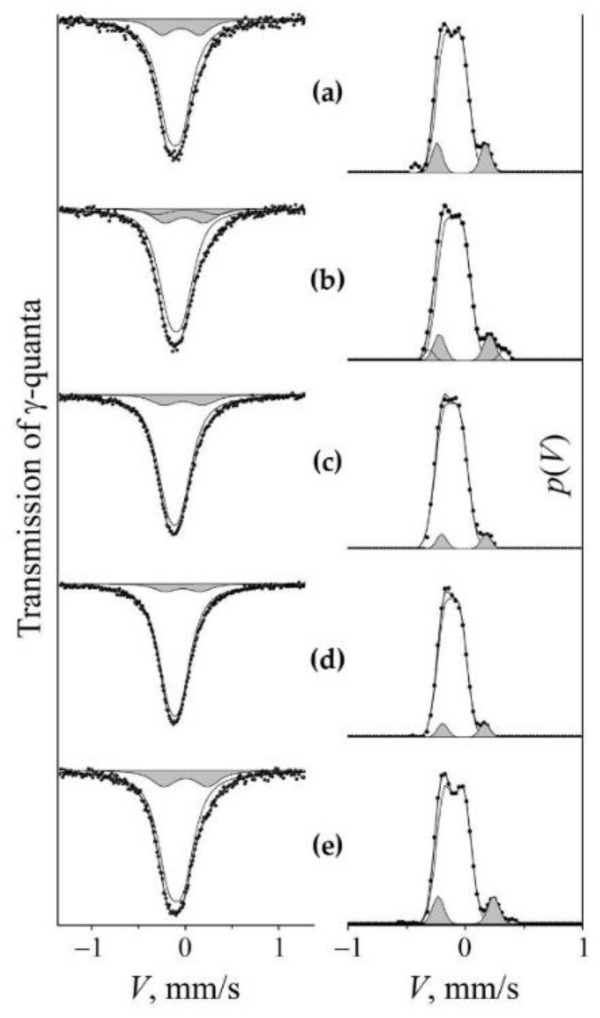
Mössbauer spectra and *p*(*V*) distributions for quenched FeMn_22_Cr_18_N_0.83_ steel with a change in the deformation temperature of the HPT of *n* = 3 rev. Treatment: (**a**)—quenching; (**b**)—HPT of *n* = 3 rev. at 298 K; (**c**)—HPT of *n* = 3 rev. at 373 K; (**d**) HPT of *n* = 3 rev. at 573 K; (**e**)—HPT of *n* = 3 rev. at 298 K, followed by annealing at 573 K, for 30 min. The component of the “nitrogenous” doublet *S_D_*_1_ is highlighted by darkening.

**Figure 6:**
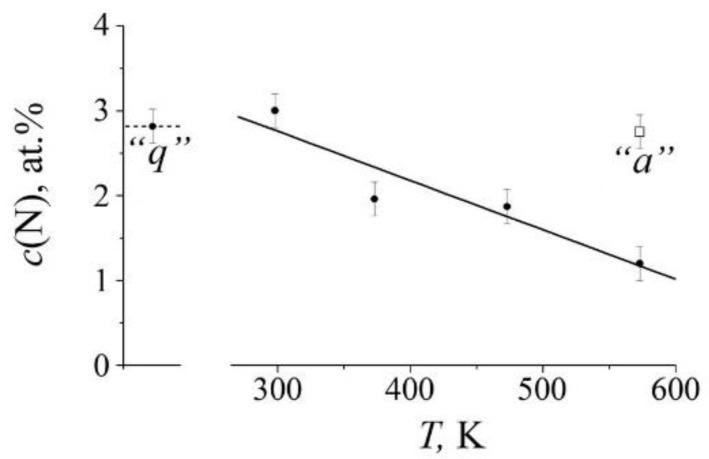
The dependence of the nitrogen content *c*(N) in the austenite matrix on the deformation temperature of the HPT of the quenched steel. The point “*q*” indicates the nitrogen content in the initial quenched steel. The point “*a*” indicates the nitrogen content after HPT of *n* = 3 at 298 K, for 30 min and subsequent annealing at 573 K, for 30 min.

The increase in the partial contribution of the “nitrogenous” *S_D_*_1_ doublet after cold (298 K) deformation of the aged sample is replaced by a decrease in the *S_D_*_1_ intensity at a temperature of 573 K, see Figure 4b2 and Table 1. In the spectrum of quenched steel, an increase in the temperature of HPT also leads to a decrease in the partial contribution of *S_D_*_1_, and the decrease begins at a sufficiently low temperature of 373 K, see Figure 5c. The decrease in the intensity of *S_D_*_1_ is regular: a change in the deformation temperature from 373 to 573 K is accompanied by a decrease in the intensity of *S_D_*_1_ from 11 to 7%. This means that in the quenched steel, in accordance with the Equation (2), in the range of deformation temperatures of HPT above 298 K, there is a decrease in the nitrogen content in the austenite-based solid solution by two times or more, see Figure 6.

#### 3.2.2. The Change in the Mössbauer Spectrum of Austenite and the Redistribution of Nitrogen Atoms Depending on the Degree of “Cold” and “Warm” Deformation ε(*n*) of Quenched and Aged Steel

**Figure 7:**
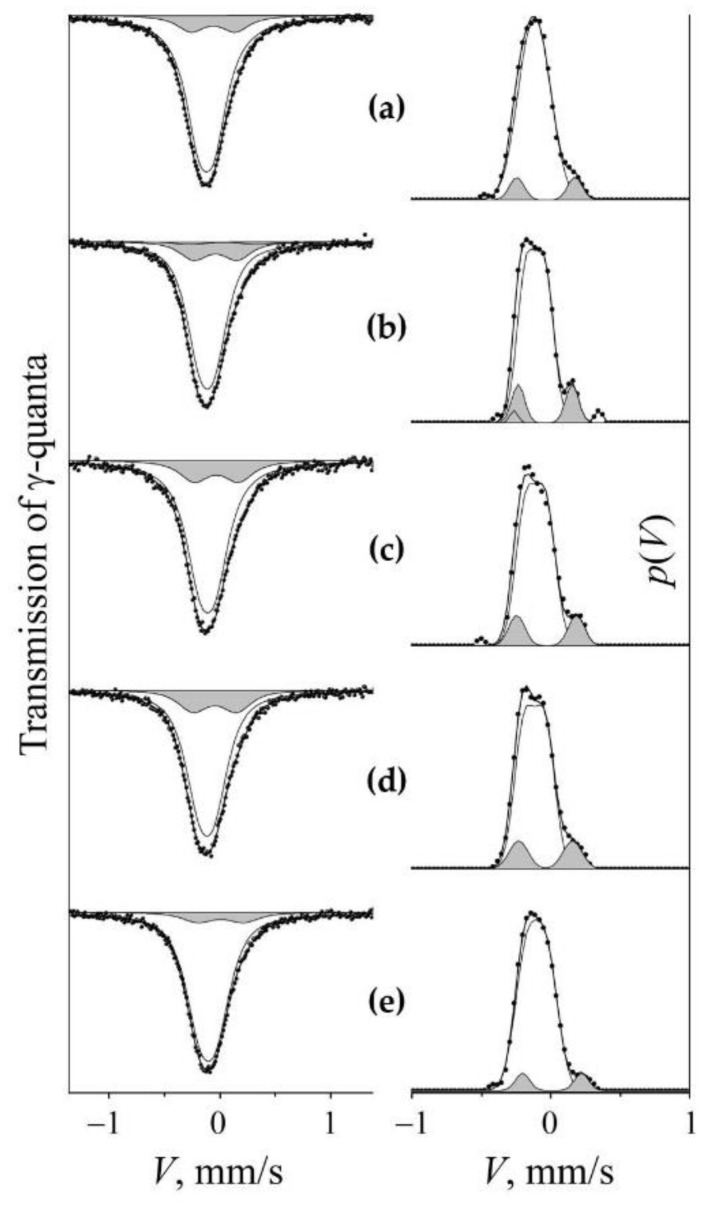
Mössbauer spectra and distributions of *p*(*V*) of FeMn_22_Cr_18_N_0.83_ pre-aged steel at 1073 K, 2 h with a change in the degree of deformation *n* (number of revolutions *n*) by HPT at 298 K. (**a**)—aging; (**b**)—*n* = 0.5 rev.; (**c**)—*n* = 1 rev.; (**d**)—*n* = 5 rev. (**e**)—spectrum and distribution of *p*(*V*) in the aged steel after HPT of *n* = 3 rev. at 573 K. The component of the “nitrogenous” doublet *S_D_*_1_ is highlighted by darkening.

**Figure 8:**
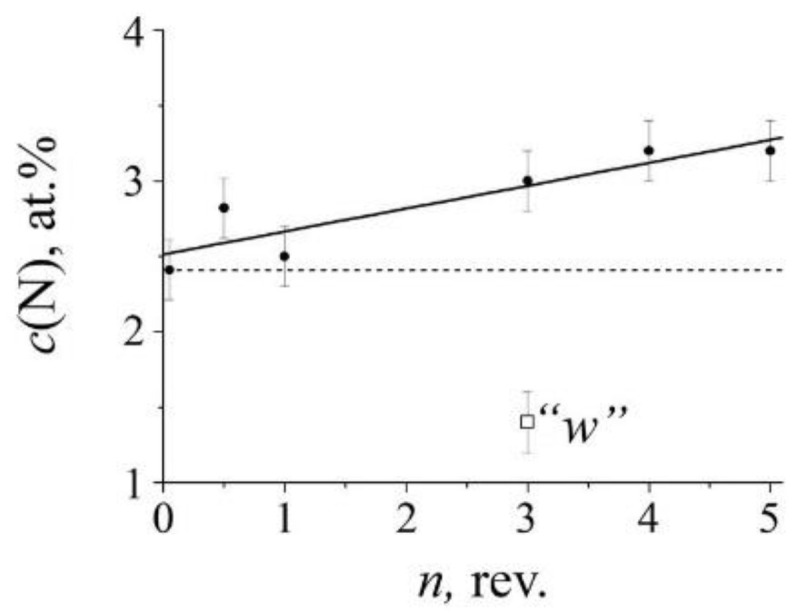
The dependence (tendency) of the nitrogen content *c*(N) in the austenite matrix on the degree of deformation *n* (number of revolutions *n*) of the pre-aged FeMn_22_Cr_18_N_0.83_ steel at 1073 K, 2 h. The dotted line indicates the nitrogen content in the aged steel; the point “*w*” indicates the nitrogen content after the HPT of *n* = 3 rev. at 298 K, for 30 min and subsequent annealing at 573 K, for 30 min.

**Figure 9:**
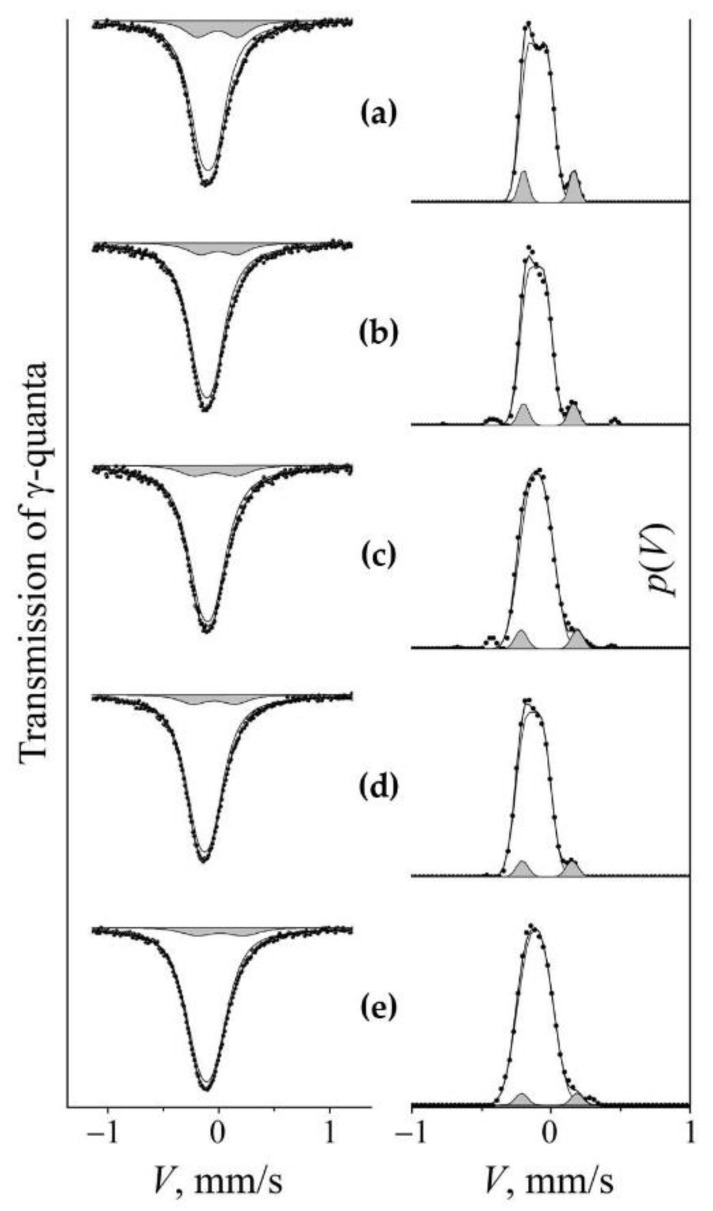
Mössbauer spectra and distributions *p*(*V*) of quenched FeMn_22_Cr_18_N_0.83_ steel with a change in the degree of deformation *n* (number of revolutions *n*) at HPT at 573 K. (**a**)—quenching; (**b**)—*n* = 0.5 rev.; (**c**)—*n* = 1 rev.; (**d**)—*n* = 3 rev.; (**e**)—*n* = 5 rev. The component of the “nitrogenous” doublet *S_D_*_1_ is highlighted by darkening.

**Figure 10:**
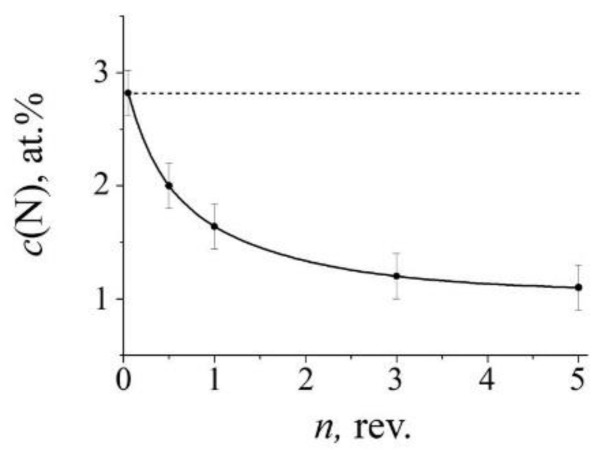
The dependence of the nitrogen content *c*(N) in the austenite matrix on the degree of deformation *n* (number of revolutions *n*) by the HPT at 573 K of the quenched FeMn_22_Cr_18_N_0.83_ steel. The dotted line indicates the nitrogen content in the quenched steel.

An increase in the degree of cold (298 K) deformation by HPT (*n*) of quenched steel does not lead to significant changes in the intensity of the “nitrogenous” doublet *S_D_*_1_, and in the case of aged steel, the growth of *n* is accompanied by an increase in *S_D_*_1_, which means an increase in the nitrogen content of *c*_N_ in austenite, see Figure 7 and Figure 8. At a deformation temperature ≥ 373 K, the growth of *n* in both quenched and aged steel causes a decrease in the intensity of *S_D_*_1_ and, accordingly, a decrease in *c*_N_, Figure 7e, Figure 9 and Figure 10. Thus, the data on the influence of the degree of deformation of the HPT in the temperature range from 298 to 373 K and above confirm the data of the Section 3.2.1 about the presence of a transitional temperature region of “dissolution–precipitation” of nitrides.

### 3.3. Mechanical Properties of Steel at Different Temperatures and Degrees of Deformation by HPT

**Figure 11:**
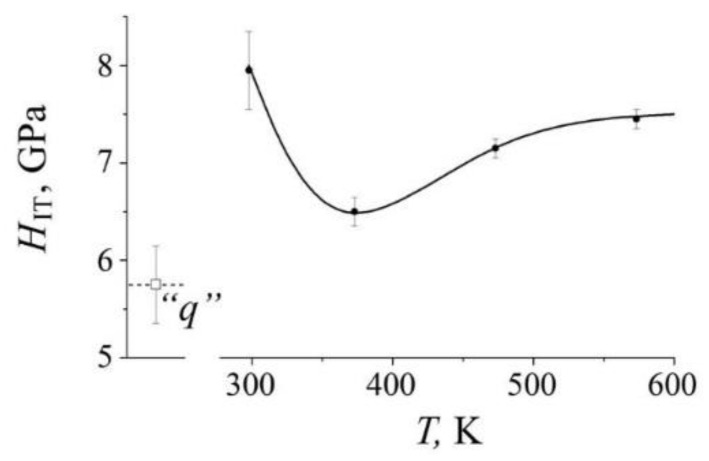
The dependence of the hardness *H*_IT_ on the deformation temperature of HPT (*n* = 3 rev.) of the quenched FeMn_22_Cr_18_N_0.83_ steel; the point “*q*” corresponds to the hardness of the initial quenched sample.

**Figure 12:**
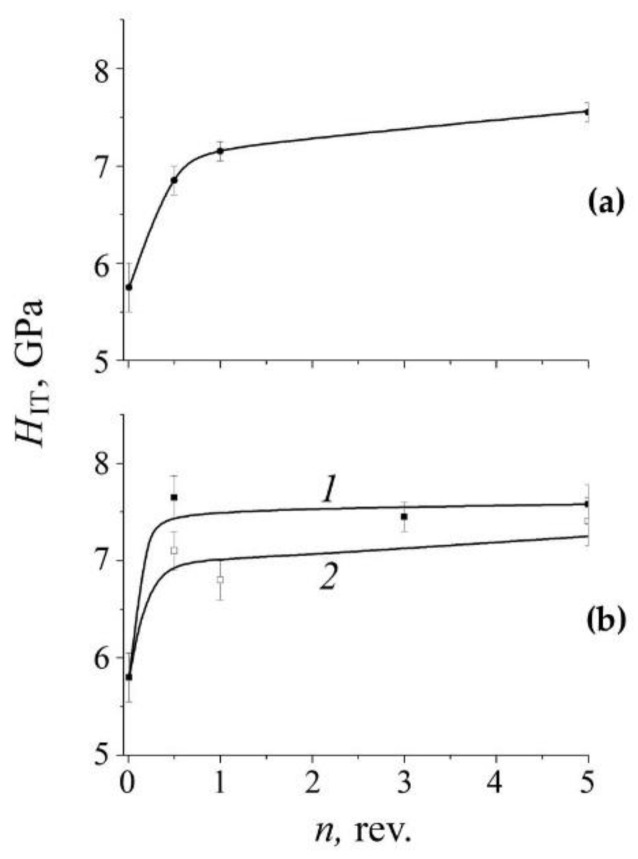
The dependence of the hardness *H*_IT_ on the degree of deformation *ε* (number of revolutions *n*) of the FeMn_22_Cr_18_N_0.83_ steel (**a**) quenched at 573 K and (**b**) aged at 1073 K, for 2 h: (*1*)—HPT-deformed at 573 K; (*2*)—HPT-deformed at 298 K.

Figure 11 shows the dependence of the average hardness *H*_IT_ of quenched steel on the deformation temperature of the HPT with *n* = 3 rev. in the temperature range from room temperature to 573 K. It can be seen that the *H*_IT_ curve has a feature that consists in a change in the temperature dependence from a decrease to an increase near the temperature point 373 K (Figure 11). Moreover, the effect of increasing *H*_IT_ in quenched steel at a temperature of 573 K increases with increasing degree of deformation, see Figure 12a. The result of an increase in hardness with an increase in the temperature and degree of deformation is confirmed in experiments on aged steel, see Figure 12b.

## 4. Discussion

### 4.1. The Relationship of the Kinetics of Nitrogen Redistribution with the Structure and Hardness of Steel at SPD

The Mössbauer analysis of the results on the increase in *c*_N_ after cold (298 K) deformation of aged samples was demonstrated earlier in experiments on the HPT and friction action on the austenitic steels FeCr_22_Mn_1.4_N_1.24_ [9] and the studied FeMn_22_Cr_18_N_0.83_ [8]. This increase is a consequence of the dissolution of nitride particles during mechanical nitrogen doping of the FCC matrix. Similar processes of dissolution of disperse particles of the second phases (intermetallides, carbides, oxides, and nitrides) in metal matrices induced by cold SPD were observed earlier [27]. The change in the direction of nitrogen redistribution during deformation of aged steel FeMn_22_Cr_18_N_0.83_ to the opposite direction with an increase in the deformation temperature suggests the presence of the formation process of particles that competes with non-equilibrium processes of their mechanical dissolution. In addition, the data on the inversion in the kinetics of nitrogen redistribution in the steel matrix in a relatively narrow range of deformation temperatures indicate the presence of a critical region in the mechanism and kinetics of structural-phase transitions in steel at SPD.

The occurrence of dynamic aging with an increase in the temperature of the SPD, and concurrent competition between dynamic aging and non-equilibrium disordering, was previously shown on substitution alloys: binary Fe–Cr, Fe–Mn, and Fe–Ni, as well as triple Fe–Ni–*Me*(Ti, Al) alloys [11,12,28]. For the Fe–Ni–*Me*(Ti, Al) alloys, the kinetics of mechanical alloying at HPT and rolling in the temperature range from 80 to 573 K was described. The change in the content of the alloying element (Ni) in the alloy matrix was described depending on the degree of deformation *ε* using the Equation (1). The change in the nickel content in the matrix occurred due to the dissolution of the intermetallic γ’ (Ni_3_Ti, Ni_3_Al) phase in a Fe–Ni matrix of FCC crystal lattice [12]:

(3) $$\Delta c=k_{1}\left(\varepsilon -\varepsilon_{\mathrm{cr}}\right)+f\left(T-T_{\mathrm{cr}}\right)$$

Here, the first term in the equation is responsible for the content of the alloying element at a given true deformation *ε* without taking into account the compensating “thermal additive”, and *ε*_cr_ is the minimum deformation at which mechanical alloying is realized [29]. The second term is responsible for the thermally activated component of the relaxation process, where *T*_cr_ is the temperature above which the competing processes of dynamic aging are activated to become concurrent. The second term can be written in the form [12]:

(4) $$f\left(T-T_{\mathrm{cr}}\right)=k_{2}D,$$

where *D* is the temperature-dependent coefficient of the diffusion of the chemical elements of alloying.

As it follows from the data of Mössbauer spectroscopy, the critical region in the kinetics of nitrogen redistribution at SPD depends on both the temperature and the degree of deformation. In accordance with the results of the experiment on cold (298 K) HPT deformation of the aged steel FeMn_22_Cr_18_N_0.83_, the first term in Equation (4) increases its contribution to the dissolution of cellular decomposition products with an increase in the number of revolutions *n*, see Figure 7 and Figure 8. The second term—which is responsible for the competing process of solid solution decomposition when a certain critical temperature range is reached,—also increases with an increase in *ε*(*n*), see Figure 9 and Figure 10.

According to the TEM data, the temperature limit detected by the Mössbauer method, above which the nitrogen content decreases in a solid solution based on austenite, is characterized by a transformation of the structure, which is resulting in the complete dissolution of cellular decomposition products and the formation of submicrocrystalline austenite with disperse nano-scale nitrides, see Figure 1, Figure 2 and Figure 3. At the same time, as it follows from the Mössbauer analysis, after deformation at 573 K, the degree of decomposition, i.e., the amount of nitrogen in a solid solution of austenite, both in the quenched and the aged steel,—approaches the value characteristic of complete decomposition of steel after artificial annealing at 1073 K, for 30 h, see Table 1. Thus, based on the TEM data, it can be concluded that an increase in the temperature of the HPT deformation accelerates both the processes of dissolution of cellular decomposition products and the formation of secondary nitrides. However, at the same time, according to the data of Mössbauer spectroscopy, the process of nitrogen release from the matrix prevails when the deformation temperature increases to 573 K.

The critical region of structural-phase transitions is determined by the parameters of the SPD, namely, temperature, rate, and degree of deformation [12], and also depends on the pretreatment of steel affecting the morphology of the decomposition products, as is shown by the example of intermetallides [27]. In for the quenched steel, the critical temperature range, characterized by a decrease in the nitrogen content in the solid solution, occurs already at 373 K in the case of *n* = 3 rev., and at 573 K, the effect of lowering the nitrogen concentration in the matrix occurs at *n* = 0.5 rev., see Figure 6 and Figure 10. In for the aged steel, the critical temperature region in the kinetics of effective mechanical alloying, characterized by a decrease in *c*_N_, occurs at the higher temperatures and degrees of deformation. Thus, the data on the Mössbauer spectroscopy and TEM on the position of the critical region of the inversion of nitrogen redistribution indicate a nonlinear dependence of the kinetics of mechanical alloying realization on the parameters of the SPD.

The presence of a critical region in the mechanism of the nitrogen dissolution induced by SPD is confirmed by the behavior of the mechanical properties of the steel. This can be seen in Figure 11 by the curve of the dependence of the hardness *H*_IT_ of quenched steel after HPT in the temperature range from room temperature to 573 K. It is obvious that there is a critical region in the dependence of hardness on the deformation temperature, which consists in a decrease in *H*_IT_ in the range from 298 to 373 K and a subsequent increase in *H*_IT_ at deformation temperatures above 373 K. The peculiarity of the *H*_IT_ dependence on temperature correlates with the Mössbauer data on the beginning of the deformation-induced decrease in the nitrogen content in the austenite solid solution, see Figure 6. The increase in the hardness of steel with an increase in temperature (above 373 K) and the degree of deformation *ε*(*n*) is consistent with the Mössbauer and TEM data on (i) the increase in the degree of decomposition of the solid solution and (ii) the formation of secondary nanocrystalline nitrides. The contribution of dynamic aging to the increase in hardness is confirmed by the results on aged steel in the form of a relative increase in *H*_IT_ in the case of “warm” deformation, Figure 12b. The influence of the degree of HPT deformation at 298 K on the *H*_IT_ of quenched and aged samples is explained by (i) the saturation of the structure with linear defects and (ii) the fragmenting of cellular decomposition products [30]. Under the conditions of “warm” deformation by the HPT at temperatures near 373 K, the dislocation number density decreases due to the processes of return and the initial stages of annealing of defects. With a further increase in the deformation temperature (above 373 K), the predominant contribution to the hardening is made by the mechanism of dispersion hardening in the form of the formation of secondary nanocrystalline nitrides [31]. Thus, when the temperature of HPT deformation changes from 298 to temperatures above 373 K, it leads to the formation of a submicrocrystalline structure—from the state of an austenite solid solution supersaturated with nitrogen to an austenite composite reinforced with nanocrystalline nitrides.

### 4.2. The Nature of Dynamic Decomposition Processes in Steel under SPD

Data on the increase in the degree of decomposition of a solid solution of FeMn_22_Cr_18_N_0.83_ steel with an increase in the number of revolutions *n* under conditions of “warm” (above 373 K) deformation by HPT indicate the dynamic nature of the processes leading to a change in the mechanism of nitrogen redistribution. Structural-phase transitions in the critical temperature range are characterized by accelerated kinetics. The duration of action of the accelerated SPD process is from 1.7 min to 17 min with an increase in *n* from 0.5 rev. to 5 rev. with a speed of anvil rotation of 0.3 rpm. The accelerated kinetics is confirmed by the almost complete disintegration of the solid solution of quenched steel after HPT at 573 K during the exposure time of 10 min (3 revolutions of anvils at a speed of 0.3 rpm). For comparison, thermal artificial annealing for 10 min and 30 min at 573 K of quenched or pre-deformed (by HPT at 298 K) steel does not lead to visible changes in the spectrum, that is, a noticeable release of nitrogen from the state of its being in a solid solution of austenite, see Figure 6, point “*a*”. Previously, the accelerated kinetics of the SPD-induced ordering and decomposition processes in substitution alloys was shown in comparison with the radiation-accelerated ordering processes in Fe–Cr and Fe–Ni alloys [11], as well as intermetallic aging in Fe–Ni–*Me*(Ti, Al) alloys under irradiation with high-energy electrons [32]. In the interstitial alloys, the acceleration of the decomposition kinetics under “warm” (573 K) deformation by HPT and electron irradiation was shown to have occurred in carbon-supersaturated Fe–Ni–C austenitic alloys [13].

During “warm” deformation, the factors affecting (i) the accelerated dissolution of primary perlite-like cellular decomposition products and (ii) the formation of secondary nano-scale nitrides are the formation of a developed network of intergranular boundaries and the saturation of the structure with linear and point defects [11,16,33]. The lowering of the nitrogen content in the metal matrix is caused by its release to the boundaries of nanoparticles in the form of segregations [34] and extremely disperse secondary nitrides [8,9,10]. It should be specially noted that the main condition for realization of an accelerated dynamic aging is the continuous generation of mobile point defects (during a few minutes exposure to SPD). The generation of vacancy complexes responsible for increasing the mobility of substitution elements in this case, chromium and manganese atoms is shown in [33]. The experimental confirmation of the formation of a high concentration of vacancy complexes, close to the concentration at pre-melting temperatures, was the work on the SPD of copper using ECAP and HPT methods of deformation [35,36]. The high diffusion mobility of nitrogen at low temperatures [3] obviously contributes to relaxation along the path of solid solution decomposition. The growth of *H*_IT_ of quenched steel at temperatures of HPT above 298 K is explained by the SPD-induced mechanism of dispersion hardening with the release of numerous nano-crystalline nitrides.

## 5. Conclusions

Using Mössbauer spectroscopy, TEM, and mechanical tests, anomalous structural-phase transitions in the high-nitrogen chromium-manganese austenitic steel FeMn_22_Cr_18_N_0.83_ were studied under shear HPT deformation with *n* from 0.5 rev. to 5 rev., in the temperature range from 298 K to 573 K. It is established that as a result of the SPD by the HPT method, in a relatively narrow “transition” temperature range (from room temperature to 373 K and above), the direction of nitrogen redistribution changes from an increase in nitrogen content in a solid solution at “cold” (298 K) deformation to a decrease at “warm” (373–573 K) deformation. The growth of the number of revolutions *n* during cold deformation by HPT increases the nitrogen content in the solid solution of austenite due to the non-equilibrium dissolution of cellular decomposition products. The growth of the number of revolutions *n* at “warm” (≥373 K) deformation, on the contrary, reduces the nitrogen content in austenite, which indicates the mechanism of dynamic decomposition. According to the TEM and Mössbauer spectroscopy data, both the dissolution of disperse nitrides and the formation of secondary nanocrystalline nitrides accelerate with an increase in the HPT deformation temperature. Above the temperature limit of the inversion, the processes of decomposition of a solid solution supersaturated with nitrogen prevail. The occurrence of an anomalous transition temperature region together with the acceleration of the kinetics of mechanical alloying during SPD is due to (i) the continuous formation of a developed network of intergranular boundaries and (ii) the saturation of the structure with linear and point defects, leading to an increase in the mobility of the atoms that the steel consists of.

The presence of a critical region in the mechanism and kinetics of structural-phase transitions induced by SPD is manifested in the abnormal behavior of the mechanical properties of steel at temperatures above room temperature. An irregular change in the *H*_IT_ consists in its increase at cold (298 K) deformation, followed by (i) a decrease in the temperature range from 298 K to 373 K and (ii) a subsequent increase at temperatures above 373 K. The increase in hardness at temperatures above 373 K is explained by the mechanism of dispersion hardening due to dynamic aging of steel.

Changing the temperature conditions of the SPD from cold to “warm” HPT deformation allows changing the submicrocrystalline structure of FeMn_22_Cr_18_N_0.83_ steel from the state of a solid solution supersaturated with nitrogen to an austenite-based composite reinforced with secondary nano nitrides. The results using HPT can be used to analyze practically important methods of SPD, for example: deformation-driven metallurgy [37], equal-channel angular pressing [38] and frictional action [6,8].

## Figures and Tables

**Table 1:** Hyperfine parameters and integral intensities of FeMn_22_Cr_18_N_0.83_ steel subspectrums in the initial quenched and aged at 1073 K, for 2-h and 30-h states and after HPT deformation (*n* = 3 rev.) at 298 and 573 K.

Treatment	*D*0			*D*1			*D*2		
	*I*_S_, mm/s	*Q*_S_/2, mm/s	*S_D_*_0_, %	*I*_S_, mm/s	*Q*_S_/2, mm/s	*S_D_*_1_, %	*I*_S_, mm/s	*Q*_S_/2, mm/s	*S_D_*_2_, %
Quenching	–0.11	0.07	84	–0.05	0.20	16	–	–	–
HPT at 298 K	–0.10	0.07	81	–0.02	0.21	17	0.01	0.32	2
HPT at 573 K	–0.11	0.06	91	–0.03	0.20	7	–	–	–
Aging at 1073 K, 2 h	–0.11	0.07	86	–0.05	0.20	14	–	–	–
HPT at 298 K	–0.11	0.07	81	–0.03	0.20	17	0.36	0.31	2
HPT at 573 K	–0.10	0.07	92	–0.01	0.21	8	–	–	–
Aging at 1073 K, 30 h	–0.12	0.06	90	–0.05	0.19	10	–	–	–
HPT at 298 K	–0.11	0.07	85	–0.03	0.20	15	–	–	–
HPT at 573 K	–0.11	0.06	91	–0.05	0.19	8	–	–	–

## Data Availability

Not applicable.
